# Protective effects and mechanism of puerarin targeting PI3K/Akt signal pathway on neurological diseases

**DOI:** 10.3389/fphar.2022.1022053

**Published:** 2022-10-24

**Authors:** Qian Wang, Zi-Nuo Shen, Shu-Jing Zhang, Yan Sun, Feng-Jie Zheng, Yu-Hang Li

**Affiliations:** School of Traditional Chinese Medicine, Beijing University of Chinese Medicine, Beijing, China

**Keywords:** PI3K/Akt signal pathway, puerarin, neurological diseases, natural products, nerve protection

## Abstract

Neurological diseases impose a tremendous and increasing burden on global health, and there is currently no curative agent. Puerarin, a natural isoflavone extracted from the dried root of Pueraria montana var. Lobata (Willd.) Sanjappa and Predeep, is an active ingredient with anti-inflammatory, antioxidant, anti-apoptotic, and autophagy-regulating effects. It has great potential in the treatment of neurological and other diseases. Phosphatidylinositol 3-kinases/protein kinase B (PI3K/Akt) signal pathway is a crucial signal transduction mechanism that regulates biological processes such as cell regeneration, apoptosis, and cognitive memory in the central nervous system, and is closely related to the pathogenesis of nervous system diseases. Accumulating evidence suggests that the excellent neuroprotective effect of puerarin may be related to the regulation of the PI3K/Akt signal pathway. Here, we summarized the main biological functions and neuroprotective effects of puerarin *via* activating PI3K/Akt signal pathway in neurological diseases. This paper illustrates that puerarin, as a neuroprotective agent, can protect nerve cells and delay the progression of neurological diseases through the PI3K/Akt signal pathway.

## 1 Introduction

With the aging of the population and the increase in life expectancy, the prevalence of neurological diseases is increasing, causing a huge health burden worldwide (Charlson et al., 2019). Including Alzheimer’s disease (AD), Parkinson’s disease (PD), traumatic brain injury (TBI), stroke, intracerebral hemorrhage (ICH), epilepsy, and other neurological diseases, affecting about one billion people worldwide, involving people of all ages, races and socioeconomic status (Misra et al., 2018). Neurological disorders have become the second leading cause of death in the world (Evans-Lacko et al., 2018), and it is also an important cause of disability and premature death. At present, there are few drugs to treat and prevent these diseases, which makes neurological diseases have become an obvious challenge (Zhou et al., 2019). Therefore, there is an urgent need to develop new drugs for the treatment of various nervous system diseases. The variety of neurological diseases with a wide range of neuropathological features, including neurological damage, cognitive dysfunction, and motor coordination disorders, has led to complex and diverse pathogenesis and involved signaling pathways, hindering the development of new drugs. Phosphatidylinositol 3-kinase/protein kinase B (PI3K/Akt) signal pathway participates in many cellular processes such as cell proliferation, differentiation, protein synthesis, and apoptosis, and is the key mechanism for the occurrence and development of central nervous system diseases (Matsuda et al., 2019). Focusing on the PI3K/Akt pathway, it is of great significance to seek reliable treatments and new drugs for neurological diseases.

Chinese herbal medicine and its natural products have the advantages of multiple targets and few adverse reactions, so they have a good prospect in the treatment of nervous system diseases (Yang et al., 2017). Puerarin is an isoflavone with high bioactivity. It is a key active substance extracted from the traditional Chinese medicine Kudzu root (Gegen in Chinese), the dried root of *Pueraria montana* var. *Lobata* (Willd.) Maesen & S. M. Almeida ex Sanjappa & Predeep. Experimental and clinical studies have reported that puerarin has been widely used in the treatment of cardiovascular disease (Zhou et al., 2021), diabetes mellitus and its complications (Bai et al., 2021), cancer (Ahmad et al., 2020), osteoporosis (Cao et al., 2022), nonalcoholic fatty liver (Wang B. et al., 2019) and endometriosis (Meresman et al., 2021). In recent years, puerarin has attracted much attention because of its excellent neuroprotective effect in AD, PD, and other central nervous system diseases. The activation of the PI3K/Akt pathway is an important way for puerarin to play a neuroprotective role. In this article, we summarized the research on puerarin protecting the nervous system by regulating PI3K/Akt pathway, in order to provide significant implications for elucidating the mechanism of puerarin in treating nervous system diseases and get further promotion in clinical application.

## 2 PI3K/AKT signal pathway and the nervous system

PI3K/Akt signal pathway is one of the classical pathways to regulate the cell cycle, which plays an important role in cell division, differentiation, and survival. PI3K is an intracellular phosphatidylinositol kinase and an important anti-apoptotic regulatory factor. Protein kinase B (PKB), also known as Akt, is an intracellular serine/threonine protein kinase. A cascade reaction pathway involving multiple signaling molecules centered on PI3K and Akt is called the PI3K/Akt signal pathway, and its activation and signal transduction regulates various physiological responses of the body. In response to various growth factors and neurotrophic factors, transmembrane receptors such as receptor tyrosine kinases (RTKs) undergo autophosphorylation, causing PI3K to be activated on the plasma membrane. Activated PI3K catalyzes the production of the second messenger phosphatidylinositol-3,4,5-trisphosphate (PIP3), which in turn recruits Akt for phosphorylation (p-Akt) at the plasma membrane. p-Akt initiates downstream effectors including mammalian target of rapamycin (mTOR), glycogensynthasekinase3β (GSK3β), and actin-related proteins, and exerts a variety of mechanisms to significantly affect cell growth and survival. Among them, mTOR, as a highly conserved serine/threonine kinase, is the core downstream factor of the PI3K/Akt signal pathway, which can be activated directly or indirectly by Akt. Meanwhile, mTOR is also an activator of Akt (Long et al., 2021). After activation, mTOR can directly act on p70 ribosomal S6 protein kinases one and 2 (p70S6K1/2), eukaryotic initiation factor 4 E-binding proteins (4 E-BPs), and other proteins involved in translation regulation, thereby initiating the protein synthesis, transcription, autophagy, metabolism, cell growth and proliferation (Lipton and Sahin, 2014).

PI3K/Akt signal pathway has been proven to play an important role in the pathogenesis of neurological diseases (Matsuda et al., 2019) and is one of the most important regulatory pathways for neuronal survival, capable of regulating neurogenesis and synaptic plasticity (Manning and Toker, 2017). Activation of the PI3K/Akt pathway promotes neuronal survival in the presence of in vitro hypoxia, excitotoxic neuronal death, and in vivo ischemic neuronal death (Lai, Zhang, and Wang, 2014). Multiple studies have demonstrated significantly reduced levels of p-PI3K and p-Akt in injured brain cells. Inhibition of the PI3K/Akt pathway exacerbates ischemic neuronal death (Endo et al., 2006). Activation of PI3K/Akt signal pathway can significantly improve acute brain injury such as cerebral hemorrhage (Krafft et al., 2013; Cui et al., 2017) and subarachnoid hemorrhage (Hao et al., 2014; Xie et al., 2018). Akt is considered a therapeutic target in neurological diseases that impact neuronal survival. Excitotoxicity is a critical link between ischemia and neuronal death (Molina et al., 2021). Akt is able to inhibit the death signal BAD (Noshita et al., 2002), the forkhead transcription factor (Trotman et al., 2006), and the proline-rich Akt substrate (Saito et al., 2004) in excitotoxicity and stroke models. Akt phosphorylates and inhibits the death-signaling protein GSK-3β, which is implicated in the pathogenesis of several neurological diseases (Chigusa et al., 2017). GSK-3β is one of the direct substrates of Akt, involved in synapse formation and microglia activation, and is able to regulate a variety of biological activities such as cell metabolism, apoptosis, inflammation, and oxidative stress. Over-activated GSK-3β leads to excitotoxic neuronal damage (French and Heberlein, 2009). In addition, its over-activation can cause synaptic defects, promote the hyperphosphorylation of tau to form neurofibrillary tangles, ultimately leading to diseases such as AD (Golpich et al., 2015; Abdallah et al., 2021). p-Akt inhibits its activity by phosphorylating GSK-3β, and phosphorylation GSK-3β (p-GSK-3β) is neuroprotective. p-GSK-3β induces the accumulation of MCL-1 protein of the Bcl-2 family, and exert an anti-apoptotic effect to protect neuronal cells (Banach et al., 2022). Studies have reported that Akt is a key mediator of several aspects of neurite growth, including elongation, branching, and caliber (Read and Gorman, 2009). Akt is able to phosphorylate GSK-3β to promote neurite growth (Ooms et al., 2006). The PI3K/Akt/mTOR pathway has been shown to promote the growth and branching of hippocampal neurons and plays an important role in the development of brain structures (Chen et al., 2003). Akt is a major upstream regulator of mTOR. mTOR is involved in the entire brain function, regulating neuronal cell synaptic plasticity, myelin formation, memory and retention (Lipton and Sahin, 2014). Studies have shown that mTOR is able to increase the length and complexity of dendrites (Jin et al., 2012). mTOR also promotes axonal regeneration in the adult central nervous system (Abe et al., 2010). In addition, mTOR can regulate a variety of downstream factors involved in neuroprotection. The sterol-response binding proteins (SREBPs) are one of the transcription factors regulated by mTOR and are involved in nutrient sensing, excitotoxicity, myelination and neurodegenerative diseases (Lebrun-Julien et al., 2014). mTOR regulates the transcription and translation of hypoxia inducible factor 1α (HIF1α), which mediates mechanisms associated with stroke and neurodegenerative diseases. Recent studies have reported that Eukaryotic Elongation Factor-2 Kinase (eEF2K) is essential for the physiopathology of the nervous system. eEF2K is able to influence synaptic plasticity, memory, and regulate protein translation in dendrites. Increased eEF2K expression is observed in AD, PD and epilepsy, and its phosphorylation is reduced as a key downstream effector of mTOR (Ballard et al., 2021). In conclusion, targeting PI3K/Akt is of great value for studying the pathogenesis of neurological diseases and new drug development.

## 3 Puerarin regulates PI3K/AKT signal pathway to protect the nervous system

### 3.1 Overview of puerarin

**FIGURE 1:**
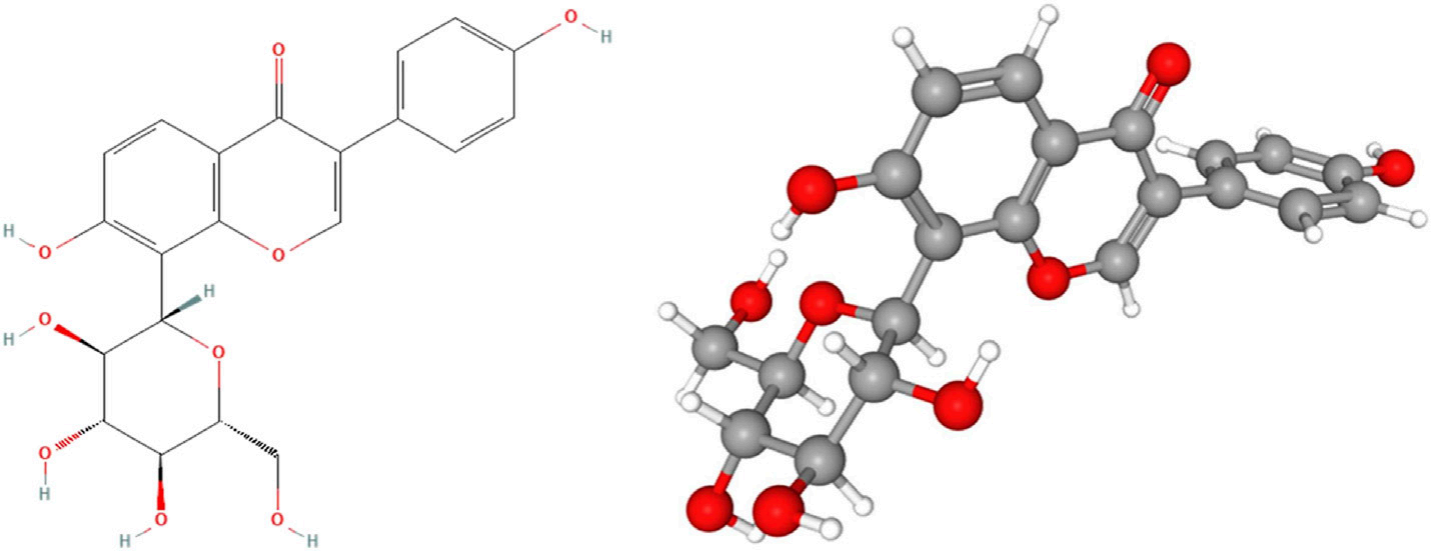
The chemical structure of puerarin. In the ball and stick model, grey, red, and white balls represent carbon, oxygen, and hydrogen atoms, respectively. (National Center for Biotechnology Information (2022). PubChem Compound Summary for CID 5281807, Puerarin. Retrieved 17 August 2022 from (https://pubchem.ncbi.nlm.nih.gov/compound/5281807).

Puerarin has the chemical structure of 7,4'-dihydroxy-8-C-glucosylisoflavone (Figure 1), molecular formula C_21_H_20_O_9_, molecular weight 416.38 (Zhou Y. X. et al., 2014). Studies have shown that puerarin can be detected rapidly and extensively in most organs such as the hippocampus, heart, and lungs after intravenous administration (Anukunwithaya et al., 2018; Zhang, 2019). Also, puerarin can be quickly eliminated. Puerarin also crosses the blood-brain barrier and is widely distributed in the hippocampus, cerebral cortex, striatum, and other regions of the brain, but at low levels (Kong et al., 2017). The gastrointestinal absorption and bioavailability of puerarin are poor. The absorption in both the stomach and intestine of rats was 20% (Chen et al., 2018), and the absolute oral bioavailability in rats (5 mg/kg, 10 mg/kg) was 7% (Anukunwithaya et al., 2018). Daidzein is the major hydrolytic metabolite of puerarin (Jung et al., 2014), which was formed by cytochrome P450 proteins in the liver microsomes (Wen et al., 2008). Glucuronoside is the main metabolite of puerarin, which is excreted in the urine and feces (Anukunwithaya et al., 2018). UDP-glucuronosyltransferase 1A1 is the principal enzyme responsible for puerarin metabolism in human liver microsomes (Luo et al., 2012). The toxicity of puerarin in experimental animals is low, but its toxicity has yet to be re-evaluated in clinical studies (Zhang, 2019). Although puerarin is easy to extract, its chemical structural properties result in poor solubility and permeability, resulting in its low oral bioavailability (Yu et al., 2022). Therefore, it is not feasible to increase the bioavailability by increasing the dose alone in clinical application, but it will increase the toxic side effects of the drug. At present, injection is the main mode of administration of puerarin (China Food and Drug Administration, http://app1.sfda.gov.cn/datasearchcnda/face3/dir.html). In order to improve the bioavailability of puerarin injections, the formulation must be supplemented with polyvinylpyrrolidone (PVP) or high concentrations of propylene glycol. However, this has consequently led to adverse effects such as fever and vascular irritation, limiting its widespread clinical use. To overcome this limitation, microemulsions and self-microemulsifying drug delivery systems (Zhang, 2019), dendrimers (Gu et al., 2013), nanoparticle carriers (Chen et al., 2019), nanocrystals (Xiong et al., 2019) and other drug delivery systems were used to improve the bioavailability and brain targeting of puerarin. In addition, structural biotransformation of puerarin using sucrose amylosucrase (Ding et al., 2022), microbial and free enzymes (Liu B. et al., 2016) was also able to improve the bioavailability of puerarin. All of the above are of great value in advancing the research and application of puerarin.

Up to now, puerarin has been widely proven to have pharmacological activities such as vasodilator, anti-inflammatory, antioxidant, anti-apoptotic, regulating autophagy, anti-insulin resistance, anti-cancer, *etc.* It has protective effects on cell damage caused by pathological factors and has shown good efficacy in the treatment of various diseases (Ma et al., 2022). Clinically, puerarin has been approved by China Food and Drug Administration as a vasodilator in the treatment of patients with coronary heart disease and hypertension (Huang et al., 2021). Puerarin has significant anti-inflammatory and antioxidant effects. It has been shown that puerarin reduced the mRNA and protein levels of Tumor necrosis factor-α (TNF-α), interleukin 6 (IL-6), and IL-1β in a mouse model of myocardial ischemia/reperfusion (MI/R) injury, inhibited NLRP3 inflammasome and protected the heart (Wang et al., 2020). Puerarin can significantly reduce reactive oxygen species (ROS) production, while excessive ROS can activate downstream apoptotic factors such as cytochrome C and apoptosis-inducing factors, and induce apoptosis (Liang et al., 2019). Puerarin can reduce ROS by increasing the gene expression of MnSod and Gpx-1, which are scavengers of ROS. It is also possible that puerarin can reduce the levels of lipid peroxidation product mitochondrial malondialdehyde (MDA) and increase superoxide dismutase (SOD) by reducing the production of ROS. Atherosclerosis is a pathological process characterized by a chronic inflammatory response, which is closely related to the inflammatory response of vascular endothelial cells. Puerarin modulates mitochondrial function in lipopolysaccharide-stimulated human umbilical vein endothelial cells, inhibits the expression of inflammatory factors and oxidative stress damage, increases autophagy and mitochondrial antioxidant capacity, and is considered to be a natural antioxidant for the treatment of atherosclerosis (Liang et al., 2019). Puerarin significantly increased the levels of antioxidant markers such as superoxide dismutase, glutathione peroxidase, and catalase in serum and liver of NAFLD, and decreased the levels of TNF-α, IL-18, and IL-1β (Zhou J. F. et al., 2022). Puerarin also mediates hepatoprotective effects by inhibiting oxidative stress and inflammation through the microRNA (miR)-34a-5p/Sirt1 axis (Hao et al., 2022). The efficacy of puerarin against preeclampsia is also related to its anti-inflammatory effect (Guo et al., 2022). In addition, puerarin also reduces pro-inflammatory factors in the chronic unpredictable mild stress (CUMS) rat model of depression *via* the gut-brain axis, and increases the level of anti-inflammatory factors, thereby achieving an antidepressant effect (Song et al., 2022). Recently, Zeng et al. (2022) also explored the anti-inflammatory mechanism of puerarin, suggesting that the anti-inflammatory effect of puerarin may be achieved by regulating multiple metabolic pathways and metabolites related to ferroptosis. Puerarin can improve diabetes and a series of complications by anti-inflammation and reducing oxidative stress. At the same time, it can also lower blood glucose, improve insulin resistance, and protect pancreatic β-cells through anti-apoptotic effects (Chen et al., 2018; Xu et al., 2021). Based on these diverse pharmacological effects, puerarin has been confirmed to have good anticancer potential and can exert therapeutic effects on cancer by blocking the cell cycle, inhibiting cell migration, inducing apoptosis, and regulating autophagy (Ahmad et al., 2020; Murahari et al., 2020). Puerarin also promotes the proliferation and differentiation of rat primary osteoblasts and human osteoblastic MG-63 cells by altering cell cycle distribution (Zhang et al., 2007; Wang et al., 2013), In contrast, puerarin inhibits vascular smooth muscle cell proliferation in the diabetic setting (Zhu et al., 2010).

### 3.2 Puerarin and PI3k/Akt signal pathway

Nevertheless, the potential mechanisms and direct targets for the diverse therapeutic effects of puerarin remain unclear. Many studies have pointed out that the activation of the PI3K/Akt signal pathway may be a key pathway for puerarin to protect the survival of various cells. Embryonic stem cells are the best platform for the research and screening of new drugs (Xi et al., 2010). Yin et al. (2015) found that puerarin could down-regulate the transcription level of RE1 silencing transcription factor (rest), a key factor for maintaining the self-renewal of embryonic stem cells, and up-regulate miR-21, a downstream target of rest, by activating PI3K/Akt pathway, thereby inhibiting the self-renewal of murine embryonic stem cells and turning them into endoderm and ectoderm differentiation. Endothelial-mesenchymal transition (EndMT) is closely related to cardiac fibrosis. Puerarin can inhibit EndMT and improve cardiac fibrosis. It was shown (Li et al., 2020) that puerarin alleviates HO-stimulated EndMT in human coronary artery endothelial cells (HCAECs) by inhibiting ROS and activating PI3K/Akt pathway. Chen et al. (2022) conducted a study using two animal models of pulmonary arterial hypertension (PAH), a rat model of monocrotaline (MCT)-induced PAH and a mouse model of hypoxia-induced hypoxic pulmonary hypertension (HPH). They confirmed that puerarin can effectively improve the structural and functional abnormalities of the pulmonary artery and right ventricle, and restore the PI3K/Akt pathway downregulated by MCT and hypoxia. Wang et al. (2022) treated cigarette smoke extract (CSE)-stimulated human bronchial epithelial cells (HBECs) with different concentrations of puerarin. The significant efficacy of puerarin in reducing ROS content and apoptosis was confirmed. It was also found that puerarin inhibited mitochondrial autophagy and bronchial epithelial cell apoptosis by activating PI3K/Akt/mTOR pathway and down-regulating DRP1 and FUN14 domain protein 1 (FUNDC1) expression. In diabetes, apoptosis is the predominant form of pancreatic β-cell death. Puerarin significantly inhibited CoCl2-induced pancreatic β-cell apoptosis and reduced ROS production and pro-apoptotic Bcl-2-associated X protein (Bax), and upregulated Sod2 and Gpx1. It is suggested that puerarin can act directly on pancreatic β-cell through anti-apoptotic and antioxidant effects by activating the PI3K/Akt pathway (Li et al., 2014). Puerarin rapidly activated Akt in mouse insulinoma β-cells (MIN6) and also restored the diminished p-Akt induced by CoCl2. Puerarin improved insulin resistance also by activating PI3K/Akt pathway (Xu et al., 2021). In terms of anti-osteoporosis, the mechanism by which puerarin promotes proliferation and differentiation of rat primary osteoblasts and human osteoblastic MG-63 cells is also achieved by increasing Akt basal phosphorylation levels in a PI3K-dependent manner (Zhang et al., 2007; Wang et al., 2013). Anti-osteoclast apoptosis is usually a key target for the prevention of glucocorticoid-induced osteoporosis. The PI3K/Akt pathway also mediates the inhibition of glucocorticoid-induced apoptosis in human fetal osteoblastic cells (hFOB1.19) by puerarin Yu et al. (2015). The PI3K/Akt pathway can be activated in a variety of human cancers, affecting the activity of transcriptional regulators. Elevated expression of its downstream factor mTOR often suggests a poor prognosis of cancer (Ahmad et al., 2020). In cancer diseases including non-small cell lung cancer (Hu et al., 2018), bladder cancer (Jiang et al., 2018), mantle cell lymphoma (Gan and Yin, 2015), chondrosarcoma (Huang et al., 2017), and other cancer diseases, puerarin was able to downregulate their PI3K, p-Akt, and mTOR levels, induce autophagy, inhibit tumor cell proliferation, and increase apoptosis. It can be seen that puerarin may have a bidirectional regulation of the PI3K/Akt pathway. In addition, the mechanisms by which puerarin inhibited apoptosis in human papillomavirus-positive HeLa cervical cancer cells (Jia et al., 2019) and ameliorated NAFLD induced by high fat and high sucrose diet in mice (Wang S. et al., 2019) were also closely related to PI3K/Akt/mTOR pathway.

Puerarin is also a natural phytoestrogen. Because of its estrogenic activity, puerarin can regulate the apoptosis of MG-63 cells through the estrogen receptor-dependent PI3K/Akt pathway (Wang et al., 2013) and inhibit the oxidative injury of Mouse hepatoma Hepa1c1c7 and HepG2 cells (Hwang and Jeong, 2008). In addition, puerarin also phosphorylated eNOS at the Ser1177 site of the reductase structural domain in EA. hy926 human endothelial cells to produce NO, which in turn protected endothelial cells (Hwang et al., 2011).

In conclusion, PI3K/Akt signal pathway is the key mechanism by which puerarin exerts protective effects on various cells. A review of the research progress of puerarin protection against neurological diseases using the PI3K/Akt signal pathway as the target is valuable to elucidate the mechanism of puerarin protection against neuronal cells.

### 3.3 Puerarin improves neurological diseases by activating PI3K/Akt signal pathway

**FIGURE 2:**
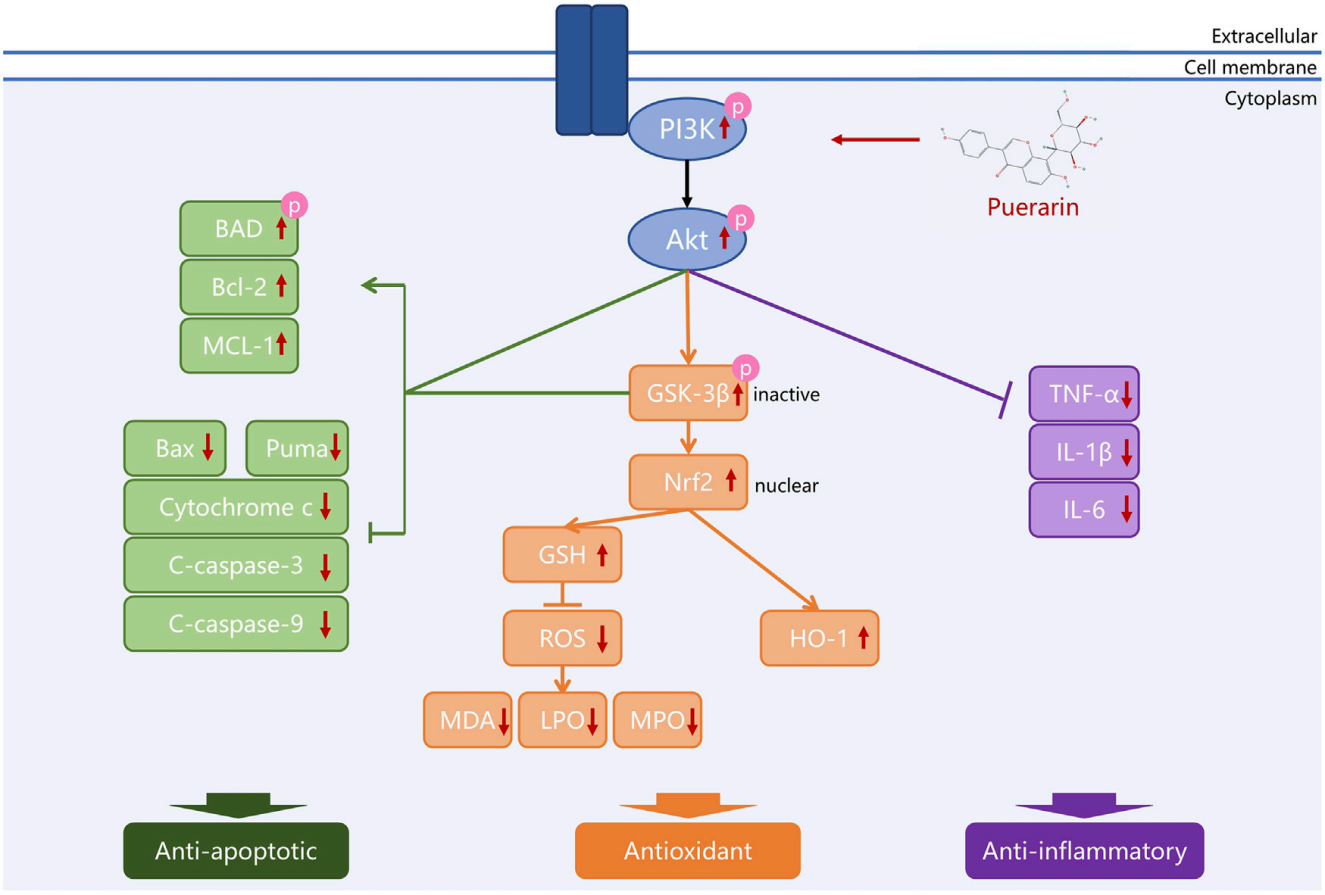
Puerarin protects neurons by regulating the PI3K/Akt pathway. Different colors are used to represent that puerarin protects neurons through anti-apoptosis, antioxidation, and anti-inflammation, which are closely related to the regulation of the PI3K/Akt pathway by puerarin.

Puerarin has potent neuroprotective effects due to its excellent anti-inflammatory, antioxidant, and anti-apoptotic properties (Yu et al., 2022). Recent studies have provided much evidence suggesting that puerarin may ameliorate neurological disorders by activating the PI3K/Akt signal pathway (Table 1 and Figure 2).

**TABLE 1:** Neuroprotective effects of puerarin on neurological diseases *via* modulating PI3K/Akt signal pathway.

Diseases	Cells/Animal	Doses	Dissolution/Administration	Mechanism	Related targets		refs
					Up-regulation	Down-regulation	
Nerve cell injury	TNF-α-induced PC12 cells	25, 50 µM	Dissolved in DMSO at 10 mM	Activating PI3K/Akt pathway; Anti-apoptotic	Bcl-2, p-Akt (Ser473)	Enzymatic activity of caspase-3 and -9, cleaved caspase-3, Bax	Liang and Xie. (2017)
Nerve cell injury	H_2_O_2_-induced apoptosis of differentiated PC12 cells	4, 8, 16 μM	Dissolved in DMSO at 10 mM	Activating PI3K/Akt pathway; Anti-inflammatory; Antioxidant; Anti-apoptotic	p-Akt, p-BAD		Zhang et al. (2012)
Lead encephalopathy	Lead acetate-induced PC12 cells	30 μM	Unclear	Activating PI3K/Akt/GSK-3β pathway; Antioxidant	GSH, GSH/GSSG ratio, GCLc, nuclear Nrf2, ARE, p-Akt, p-GSK-3β	ROS, LPO, cytosolic Nrf2	Li et al. (2014)
AD	Aβ_25-35_-induced PC12 cell	50, 100, 200 µM	Solubilized in 1,2-propanediol	Activating PI3K/Akt pathway; Anti-apoptotic	Bcl-2, p-BAD, p-Akt	Bax, cleaved caspase-3, cytochrome c	Xing et al. (2011)
AD	Aβ_25-35_-induced BV-2 and mouse primary microglial cells	20, 100 µM	Dissolved in saline	Activating PI3K/Akt pathway; Antioxidant; Anti-apoptotic	Bcl-2, p-Akt	Bax, cleaved caspase-3, cytochrome c, ROS	Wang et al. (2014b)
AD	APP/PS1 transgenic mice	30 mg/kg/day	Oral, solubilized in 1,2-propanediol	Activating Akt/GSK-3β pathway; Antioxidant	pAkt-Ser473, pSer9-GSK-3β, GSH, HO-1	LPO, MDA	Zhou et al. (2014a)
PD	MPTP-lesioned mice; PC12 cells	50, 150 mg/kg/day; 50 μM	via i.p. Injection; Dissolved in 100μl saline containing 50% 1,3-propanediol	Activating PI3K/Akt pathway; Activating ERK1/2 pathway	TH, p-Akt, MAP2, β3-tubulin, nuclear Nrf2, p-ERK1/2, HO-1		Zhao et al. (2015b)
PD	MPTP-lesioned mice	0.04, 0.12 mg/kg/d	via i.p. Injection	Activating PI3K/Akt pathway; Antioxidant	p-Akt, DA, DOPAC, Lamp 2A, GDNF, GSH	ROS	Zhu et al. (2014)
PD	MPP^+^-induced SH-SY5Y cells	50 μM	Dissolved in DMSO	Activating PI3K/Akt pathway; Anti-apoptotic; Inhibiting programmed cell death	p-Akt	Nuclear p53, Puma, Bax, caspase-3	Zhu et al. (2012)
ICH-induced early brain injury	Collagenase IV injection-induced rat	50, 100 mg/kg	via i.p. Injection, dissolved in DMSO	Activating PI3K/Akt pathway; Anti-inflammatory;Antioxidant; Anti-apoptotic	PI3K, p-Akt, Bcl-2	Bax, cleaved caspase-3, TNF-α, IL-1β, IL-6	Zeng et al. (2021)
Ischemic stroke	I/R Rat	50, 100 mg/kg	via i.p. Injection, dissolved in 10% methyl glycol	Activating Akt/GSK-3β/MCL-1 pathway; Anti-apoptotic	p-Akt1, p-GSK-3β, MCL-1	Cleaved caspase-3	Tao et al. (2017)
Hypoxic brain injury	Hypoxia-induced NSCs	20-100 μM	Unclear	Activating PI3K/Akt pathway; Activating MEK/ERK pathway; Anti-apoptotic	miR-214, p-PI3K, p- Akt, p-MEK, p-ERK	Cleaved caspase-3, cleaved caspase-9	Wang et al. (2019a)
SCI	T8 laminectomy-induced rat	25, 50, 100 mg/kg	via i.p. Injection, dissolved in DMSO	Activating PI3K/Akt pathway; Anti-inflammatory; Anti-apoptotic	GAP-43, Bcl-2, PI3K, p-Akt (Ser473)	GFAP, OX-42, TNF-α, IL-1β, IL-6, Bax, cleaved caspase-3	Zhang et al. (2016)
TBI	Feeney weight-drop rat model	200 mg/kg	via i.p. Injection, dissolved in the vehicle (cremophor:ethanol:normal saline ¼ 1:1:4)	Activating PI3K/Akt pathway; Antioxidant	p-Akt, GSH	MDA, MPO	Wang et al. (2014b)
Epileptic	Pentylenetetrazol-induced seizures in mice	320 mg/kg	via i.p. Injection	Activating PI3K/Akt/GSK-3β pathway; Anti-inflammatory; Antioxidant	p-PI3K/PI3K, p-Akt/Akt, p-GSK-3β/GSK-3β, SOD	MDA, IL-6, TNF-α	Guan et al. (2021)

^a^ p., intraperitoneal; DMSO, dimethylsulfoxide; MPP+, 1-methyl-4-phen-ylpyridinium iodide; I/R, ischemia/reperfusion; LPO, lipid hydroperoxide; APP/PS1, amyloid precursor protein/presenilin-1; T8, the eighth thoracic segment; MPO, myeloperoxidase; MPTP, 1-methyl-4-phenyl-1, 2,3,6-tetrahydropyridine; DOPAC, dihydroxyphenylacetic acid; DA, dopamine; TH, tyrosine hydroxylase; NSCs, neural stem cells; GDNF, glial cell line-derived neurotrophic factor; GCLc, glutamate cysteine ligase catalytic subunit; MAP2, microtubule-associated protein 2.

#### 3.3.1 Nerve cell injury

PC12 cells are a differentiated cell line of rat adrenal medullary pheochromocytoma with general characteristics of neuroendocrine cells. It has been widely used in neurophysiological and neuropharmacological studies (Wang et al., 2015). Liang and Xie (Liang and Xie, 2017) used TNF-α to induce neurotoxicity in PC12 cells. It was found that puerarin significantly promoted Akt (Ser473) phosphorylation and inhibited TNF-α-induced apoptosis. Application of PI3K inhibitor LY294002 attenuated the effect of puerarin, indicating that puerarin inhibited TNF-α-induced apoptosis in PC12 cells by activating the PI3K/Akt pathway. Zhang et al. (2012) reported the effect and mechanism of puerarin on oxidative stress-induced neurotoxicity in PC12 cells. The results showed that puerarin was able to dose-dependently inhibit H_2_O_2_-induced PC12 cells from apoptosis under oxidative stress by increasing the production of p-Akt and apoptotic factor p-BAD (Bcl-2/Bcl-XL-antagonist). These protective effects were reversed by the highly specific PI3K inhibitor wortmannin, suggesting that puerarin may exert antioxidant and anti-apoptotic effects by activating the PI3K/Akt pathway. The central nervous system is the main target of lead poisoning, and neuronal oxidative stress is the key mechanism (Lu et al., 2013). Puerarin has great potential to treat lead neurotoxicity-related diseases due to its excellent antioxidant effect. It was noted (Li et al., 2014) that puerarin significantly attenuated ROS and lipid hydroperoxide levels in lead acetate-induced PC12 cells, and induced phosphorylation of Akt and GSK-3β. In addition, puerarin also elevated the level of glutathione (GSH), a cellular antioxidant, and upregulated the expression of glutamate cysteine ligase catalytic subunit (GCLc), a major rate-limiting enzyme for GSH synthesis. Puerarin induces nuclear factor erythroid 2-related factor 2 (Nrf2) nuclear accumulation, thus exerting a role in regulating GSH biosynthetic enzymes. The above protective effect of puerarin could be blocked after using LY294002. It indicates that puerarin exerts an antioxidant mechanism to ameliorate lead poisoning injury in neuronal cells, mainly through activation of the PI3K/Akt/GSK-3β pathway. In summary, puerarin exerts anti-apoptotic, antioxidant, and anti-inflammatory effects to ameliorate neuronal cell injury through activation of the PI3K/Akt pathway.

#### 3.3.2 Alzheimer’s disease

AD is a neurodegenerative disease characterized by progressive memory and cognitive impairment, and behavioral and motor disorders. The main pathological features are plaques of β-amyloid (Aβ) and neurofibrillary tangles composed of hyperphosphorylated tau. As well as extensive synaptic and neuronal loss, oxidative stress and neuroinflammation, for which there is no effective treatment (Knopman et al., 2021). More and more studies have confirmed that puerarin can effectively improve AD cognitive dysfunction through multiple mechanisms (Yu et al., 2022). Puerarin reduces Aβ deposition, decreases protein levels of hyperphosphorylated tau and its phosphorylation levels, alleviates neuroinflammation, and prevents neuronal loss (Huang et al., 2019). Puerarin can improve synaptic plasticity impairment in AD by regulating the p38 MAPK-CREB signal pathway (Liu C. et al., 2021). Increased ROS production can directly impair synaptic plasticity and cause cognitive dysfunction (Butterfield and Halliwell, 2019). Puerarin can increase the activity of oxidative stress markers glutathione peroxidase (GSH-Px) and superoxide dismutases (SOD) in brain tissue, and reduce ROS generation and the level of lipid peroxidation product malondialdehyde (MDA) (Zhao J. et al., 2015). Puerarin also alleviated A*β*_25-35_-induced PC12 cell apoptosis and increased the Bcl-2/Bax ratio (Zhang et al., 2008). PI3K/Akt pathway is one of the important mechanisms by which puerarin ameliorates AD cognitive impairment. It was shown Xing et al. (2011) that puerarin was able to activate Akt phosphorylation in A*β*_25-35_-induced PC12 cells. Meanwhile, puerarin up-regulated the levels of Bcl-2 and p-BAD, and down-regulated Bax and cleaved caspase-3. It is suggested that puerarin improves Aβ2_5-35_-induced PC12 cell apoptosis depending on PI3K/Akt pathway. Wang C. et al. (2014) used A*β*_25-35_-induced BV-2 and mouse primary microglial cells as AD models and further confirmed that the anti-apoptotic effect of puerarin on AD is dependent on PI3K/Akt signal pathway. In addition, they found that A*β*_25-35_-induced primary microglial cells exhibited disruption of mitochondrial membrane potential and increased ROS production, which was inhibited by puerarin. Zhou Y. et al. (2014) also confirmed that puerarin can activate the Akt/GSK-3β signal pathway in amyloid precursor protein/presenilin-1 (APP/PS1) mice and induce Nrf2 nuclear translocation in the hippocampus. Puerarin also down-regulated lipid hydroperoxide and MDA, and up-regulated the levels of GSH and antioxidant enzyme heme oxygenase-1 (HO-1). These results suggest that puerarin improves AD cognitive dysfunction by exerting antioxidant effects, rather than by altering soluble or insoluble A*β*_1-42_ levels. In addition, puerarin can activate PI3K/Akt/eNOS pathway to reduce ROS production and avoid neuronal death (Lu et al., 2014; Liu et al., 2019). It can be seen that puerarin has a good therapeutic prospect in improving AD cognitive dysfunction, and its mechanism is closely related to PI3K/Akt-mediated anti-apoptosis and antioxidant. However, there are still few studies on the mechanism of improvement of AD by puerarin, and more studies targeting PI3K/Akt pathway are needed in the future. This is of great interest to elucidate the mechanism of action of puerarin in improving AD.

#### 3.3.3 Parkinson’s disease

PD is the second major neurodegenerative disorder characterized by progressive loss of nigrostriatal dopaminergic neurons, manifested by bradykinesia, rigidity, or resting tremor. Like AD, there is still no effective treatment (Aborode et al., 2022). Several studies have reported that puerarin can improve PD through neuroprotection and antioxidant effects, but the specific mechanism is unclear. Nerve growth factor (NGF), a neurotrophic factor, is significantly downregulated in the substantia nigra of PD patients and inhibits neuronal function, growth, and differentiation (Ubhi et al., 2010). The PI3K/Akt signal pathway is an important mechanism regulating the neurotrophic activity of NGF (Tzeng et al., 2021). A study by Zhao J. et al. (2015) noted that puerarin significantly improved tyrosine hydroxylase levels and motor dysfunction in dopaminergic neurons of 1-methyl-4-phenyl-1,2,3,6-tetrahydropyridine (MPTP)-lesioned mice. In PC12 cells, the combination of NGF (2 ng/ml) with puerarin (50 μM) greatly enhanced NGF-promoted neurite formation and length, as well as the levels of microtubule-associated protein 2 (MAP2) and β3-tubulin. Further studies confirmed that puerarin activates PI3K/Akt and ERK1/2 pathways to promote Nrf2 into the nucleus, upregulates HO-1, and subsequently enhances NGF-induced neurogenesis. ROS is thought to be critical in mediating dopaminergic neuronal cell death. It has been suggested (Zhu et al., 2014) that puerarin can exert antioxidant effects by activating PI3K/Akt pathway, which in turn reduces ROS production, and upregulates the expression of GSH and glial cell line-derived neurotrophic factor (GDNF), thereby alleviating MPTP-induced of motor dysfunction and degeneration of dopaminergic neurons. Puerarin also ameliorated oxidative stress-induced reduction in chaperone-mediated autophagy (CMA) activity, a hallmark of PD pathogenesis (Zhu et al., 2014; Kuo et al., 2022). In addition, puerarin was able to activate the PI3K/Akt pathway in 1-methyl-4-phen-ylpyridinium iodide (MPP^+^)-induced dopaminergic SH-SY5Y cells and inhibit p53-mediated caspase-3-dependent programmed cell death (Zhu et al., 2012). In conclusion, puerarin is able to restore neurotrophic factor levels and improve motor dysfunction and dopaminergic neuronal degeneration in PD by activating the PI3K/Akt pathway. It is necessary to conduct more experimental and clinical studies.

#### 3.3.4 Intracerebral hemorrhage

ICH refers to primary cerebral parenchymal hemorrhage, a cerebrovascular disease with high disability and mortality rates worldwide, for which there is no specific and effective treatment (Zhou J. et al., 2022). A growing number of studies have shown that high-intensity oxidative stress, neuroinflammation, and neuronal apoptosis are important pathogenic mechanisms of secondary brain injury after ICH (Hu et al., 2016; Zheng et al., 2016; Lan et al., 2017). Therefore, targeting the regulation of oxidative stress, inflammatory response, and apoptosis in brain tissue is the key to the treatment of secondary brain injury after ICH. Zeng et al. (2021) showed that puerarin was able to activate the PI3K/Akt pathway after ICH, which in turn inhibited the activation of apoptotic signaling, reduced oxidative stress, and downregulated the levels of pro-inflammatory factors, ultimately relieving the early brain injury after ICH. The results showed that in an ICH rat model induced by collagenase IV injection, both 50 mg/kg and 100 mg/kg doses of puerarin significantly upregulated the expression levels of PI3K and p-Akt, thereby reducing the histological damage, cerebral edema, cerebral hematoma volume, and blood-brain barrier damage. In addition, apoptosis of brain cells was significantly increased after ICH. The production of ROS around the hematoma increased, and the activation of the NF-κB pathway caused a significant increase in the levels of various inflammatory factors. Puerarin can regulate the levels of B-cell lymphoma-2 (Bcl-2), Bax, and cleaved caspase-3, inhibit cell apoptosis, and reduce the levels of ROS and various inflammatory factors. These results confirmed that puerarin can exert anti-apoptotic, antioxidant, and anti-inflammatory effects by activating PI3K/Akt pathway to alleviate early brain injury caused by ICH. However, there are still relatively few studies on the effects and mechanisms of puerarin in improving cerebral hemorrhage, and more evidence is needed to support this.

#### 3.3.5 Ischemic stroke

Ischemic stroke, caused by rupture or occlusion of blood vessels, can cause irreversible damage to the brain. It is a common cause of death and severe disability, with millions of people worldwide suffering from ischemic stroke each year (Benjamin et al., 2019). Previous studies suggest that puerarin can exert cerebral protective effects by inhibiting apoptosis after cerebral ischemia (Xu et al., 2005). In China, puerarin injections have been widely used in the clinical treatment of patients with acute ischemic stroke. By evaluating 20 randomized controlled trials (RCTs) with 1574 participants as of 2015, Liu G. et al. (2016) suggested that puerarin improves neurological impairment after ischemic stroke. Hu et al. (2012) evaluated 15 RCTs and 1603 patients, and the results showed that puerarin was more effective than the control treatment for acute cerebral infarction. Tao et al. (2017) used a rat model of cerebral ischemia/reperfusion (I/R) as the experimental subject to investigate the mechanism of puerarin in improving ischemic stroke. The results showed that puerarin could upregulate p-Akt1 (Ser473), p-GSK-3β (Ser9), and myeloid cell leukemia-1 (MCL-1), and downregulate cleaved caspase-3, thereby improving the survival rate of neurons in cerebral cortex and hippocampus. PI3K inhibitor LY294002 was able to counteract the neuroprotective effects of puerarin mentioned above. This study provides a new idea to reveal the mechanism of puerarin in the treatment of ischemic stroke, but the way of activation of the PI3K/Akt pathway by puerarin remains to be further elucidated. In addition, the combination of puerarin and catalpol (an active monomer isolated from Rehmannia glutinosa) could protect neurovascular units (neurons, astrocytes, and brain vascular endothelial cells) by improving edema, anti-inflammation, antioxidant, and anti-apoptosis, and this protective effect was dependent on the regulation of PI3K/Akt/mTOR/HIF-1α and ERK/HIF-1α pathways (Liu et al., 2017). In conclusion, although puerarin has been widely used in the clinical treatment of acute ischemic stroke patients, its mechanism of action is still unclear. PI3K/Akt pathway may be the key mechanism for puerarin to improve ischemic stroke, which needs to be further elucidated by numerous studies in the future.

#### 3.3.6 Hypoxic brain injury

Hypoxia can cause different degrees of brain damage, especially in neonates. It is a major cause of neonatal death and disability (Kennedy et al., 2022). MiR-214 is involved in cell differentiation and apoptosis and is associated with myocardial damage (Liu S. et al., 2021; Xiao et al., 2021), hepatocellular carcinoma cell invasion (Zhao et al., 2022), and rectal cancer (Yang et al., 2021). It was shown that PI3K/Akt/mTOR pathway mediates hypoxia-induced apoptosis and autophagy (Gong et al., 2019), and regulation of miR-214 could activate PI3K/Akt/mTOR pathway to alleviate hypoxia-induced apoptosis and autophagy. To explore the role and mechanism of puerarin in ameliorating hypoxic brain injury, Wang B. et al. (2019) isolated neural stem cells (NSCs) from the hippocampus of E14 rats treated with hypoxia (an anaerobic gas mixture of 94% N_2_, 5% CO_2_, and 1% O_2_ for 8 h), and administered puerarin (20–100 μM) pretreatment. The results showed that puerarin (60 μM) significantly increased the cell viability of hypoxia-induced NSCs, and inhibited the expression of cleaved caspase-3 and -9. Puerarin relieved PI3K and Akt phosphorylation inhibited by hypoxia, while miR-214 deficiency treatment inhibited the effect of puerarin on PI3K and Akt phosphorylation. In conclusion, puerarin can activate PI3K/Akt pathway through upregulation of miR-214 and exert a protective effect against hypoxic NSCs injury through anti-apoptosis. The above study suggests that miR-214 may be a target for geranium to regulate the PI3K/Akt pathway to protect neuronal cells, but further studies are needed.

#### 3.3.7 Traumatic central nervous system injury

Puerarin has neuroprotective effects against secondary injury in acute spinal cord injury (SCI) and TBI. SCI is a traumatic injury caused by mechanical damage to the spinal cord tissue, which can undergo secondary injury such as neuronal apoptosis, glial cell activation, axonal degeneration, and ultimately lead to neurological dysfunction (Noristani, 2022). Zhang et al. (2016) found that puerarin was able to upregulate PI3K and p-Akt (Ser473), and significantly restore motor function in a rat model of SCI by exerting anti-inflammatory and anti-apoptotic effects. Puerarin can also maintain the survival and cell morphology of neurons and promote the regeneration of neurons. In addition, puerarin significantly inhibited the activation of astrocytes and microglia. It is evident that PI3K/Akt is an important mechanism by which puerarin protects against secondary injury in SCI. Primary brain injury in TBI is difficult to intervene in, but reversible secondary brain injury has critical therapeutic implications (Bramlett and Dietrich, 2007). Studies have shown that the degree of oxidative stress is closely related to the severity of secondary brain injury in TBI, and protection against secondary brain injury in TBI through antioxidants is of interest (Wang J. W. et al., 2014). Wang C. et al. (2014) demonstrated that puerarin can down-regulate MDA, Myeloperoxidase (MPO), and up-regulate GSH by activating the PI3K/Akt pathway and improve TBI-induced neurodegeneration by exerting antioxidant and anti-inflammatory effects. It can be seen that puerarin has a good therapeutic prospect in the treatment of SCI and TBI secondary injury. The PI3K/Akt pathway may be the key mechanism for this effect of puerarin in promoting neuronal survival and regeneration.

#### 3.3.8 Epilepsy

Epilepsy is caused by excessive abnormal discharge of nerve cells in the brain. Persistent seizures can lead to oxidative stress and inflammation in the brain, damage brain tissue, and cause cognitive dysfunction (Knowles et al., 2022). Puerarin is able to improve epilepsy-induced brain damage through anti-inflammatory, antioxidant, and anti-apoptotic effects, but the exact mechanism is unclear (Xie et al., 2014). Guan et al. (2021) found that puerarin significantly reduced the number, duration, and mean escape latency of pentylenetetrazol-induced seizures in mice. Further research found that puerarin improved epileptic seizures and cognitive function in mice by activating the PI3K/Akt/GSK-3β signal pathway. Although there are still few studies on the mechanism of puerarin to ameliorate epilepsy-induced brain injury, a related study targeting the PI3K/Akt pathway provides us with new ideas to understand the antiepileptic effects of puerarin.

## 4 Conclusion and perspective

The activation of the PI3K/Akt pathway may be the key pathway for puerarin to ameliorate a variety of diseases and cells; therefore, it is important to review the research progress of puerarin in protecting neurological diseases by targeting the PI3K/Akt pathway. In summary, the mechanism of action of puerarin in regulating the PI3K/Akt pathway to protect neuronal cells is complex and mutual, mainly related to antioxidant, anti-apoptotic and anti-inflammatory, but the specific molecular mechanisms involved are still unclear. Among them, the antioxidant effect of puerarin is considered the central mechanism of action and investigated deeply by many studies. Although most of the biological effects of puerarin on different cells have been shown to be related to the regulation of the PI3K/Akt pathway, there are still many limitations of the existing studies. For example, current evidence does not explain how puerarin targets activation of the PI3K/Akt pathway and the direct target of puerarin action remains unclear. Some arguments suggest that puerarin (Hwang and Jeong, 2008; Wang et al., 2013), because of its natural estrogenic activity, may interact directly with the estrogen receptor, binding to the p85 regulatory subunit of PI3K in a ligand-dependent manner, which in turn causes an Akt phosphorylation hierarchical response. Further studies on the direct interaction between puerarin and estrogen receptors are needed in the future to better explain this issue. This is a key question that needs to be addressed for many natural drugs. Further mechanistic studies might help in understanding the significance at the molecular level. Furthermore, although most studies suggest that puerarin exerts its biological activity by activating the PI3K/Akt pathway, in diseases such as cancer, hepatic fibrosis (Guo et al., 2013), and cardiac hypertrophy (Yuan et al., 2014), the therapeutic effects of puerarin are mediated by blocking the PI3K/Akt pathway. These results suggest that puerarin may be a bidirectional regulator of the PI3K/Akt pathway. This also requires more research to demonstrate and analyze. Notwithstanding the continued progress made in the treatment of neurological diseases of this natural agent, the development and application of puerarin as a novel drug still require more experimental. According to the available Pharmacokinetics studies on Puerarin, there is still room for improvement in its solubility, permeability, and bioavailability of Puerarin. To improve the bioavailability of puerarin, a number of approaches have been developed in recent years to improve the brain targeting of the drug, including the use of drug delivery systems and structural biotransformation. However, puerarin is still mainly administered by intraperitoneal injection in the current *in vivo* studies. Further research on puerarin in combination with new techniques of drug delivery is necessary. Although puerarin has been used clinically in the treatment of ischemic stroke in China, the quality of clinical studies needs to continue to improve. Also, the efficacy and mechanisms of puerarin in the treatment of other neurological disorders need to be supported by clinical studies. Finally, although the toxicity of puerarin to experimental animals is low, the existing toxicology studies are far from adequate. More and higher quality studies are needed, especially in human populations.
